# Multilevel Modelling of Individual, Community and Regional Level Factors Associated with Insecticide-Treated Net Usage among Pregnant Women in Ethiopia

**DOI:** 10.3390/healthcare10081418

**Published:** 2022-07-29

**Authors:** Kenenisa Abdisa Kuse, Teshita Uke Chikako, Reta Habtamu Bacha, John Elvis Hagan, Abdul-Aziz Seidu, Bright Opoku Ahinkorah

**Affiliations:** 1 Department of Statistics, Bule Hora University, Bule Hora P.O. Box 144, Ethiopia; abdisakenenisa40@gmail.com; 2 Wondo Genet College of Forestry and Natural Resource, Hawassa University, Hawassa P.O. Box 05, Ethiopia; teshitauke@hu.edu.et; 3 Department of Statistics, Jimma University, Jimma P.O. Box 378, Ethiopia; reta.habtamu@ju.edu.et; 4 Department of Health, Physical Education, and Recreation, University of Cape Coast, Cape Coast PMB TF0494, Ghana; 5 Neurocognition and Action-Biomechanics-Research Group, Faculty of Psychology and Sport Sciences, Bielefeld University, 33501 Bielefeld, Germany; 6 Department of Population and Health, University of Cape Coast, Cape Coast PMB TF0494, Ghana; abdul-aziz.seidu@stu.ucc.edu.gh; 7 College of Public Health, Medical and Veterinary Sciences, James Cook University, Townsville, QLD 4811, Australia; 8 Department of Estate Management, Takoradi Technical University, Takoradi P.O. Box 256, Ghana; 9 School of Public Health, Faculty of Health, University of Technology Sydney, Sydney, NSW 2007, Australia; bright.o.ahinkorah@student.uts.edu.au

**Keywords:** Ethiopia, insecticide-treated net, malaria, mosquitoes, pregnant women

## Abstract

Background: Pregnant women who are infected with malaria usually have more severe symptoms and negative health outcomes than women who are not pregnant, with higher rates of miscarriage, intrauterine demise, premature delivery, low-birth-weight neonates, and neonatal death. Based on evidence from the 2016 Ethiopia Demographic and Health Survey, this study assessed the variation in insecticide-treated nets (ITNs) usage and its associated factors among pregnant women in Ethiopia. Methods: Data from a total of 1122 women who were pregnant at the time of the survey were included in the final analysis. Bivariate and multilevel analyses were conducted. Multilevel modeling with fixed and random coefficients was used to estimate the variation in pregnant women’s ITNs usage across communities (residence areas) and regions of Ethiopia. Results: Out of the total 1122 pregnant women, 58.37% slept under a net and 41.63% did not sleep under nets. Significant variations were observed in pregnant women’s ITNs usage across communities (residence areas) and regions of Ethiopia, with between variations in pregnant women’s ITNs usage across communities (residence areas) and regions. In addition, the region, place of residence, wealth index, educational level, and age of the women as well as whether they believed that mosquito bites cause malaria were significant factors in pregnant women’s usage of ITNs. Pregnant women in Ethiopia had moderate usage of ITNs with varied risk factors at the individual, community, and regional levels. Conclusion: Based on the factors identified, there is a need to implement and/or strengthen programs (e.g., regular sensitization) that intensify high coverage of ITNs for effective malaria prevention in Ethiopia, especially among pregnant women who do not use ITNs.

## 1. Introduction

Malaria is a preventable and curable life-threatening disease caused by parasites that are transmitted from person to person through bites of infected female Anopheles mosquitoes [1]. For many countries affected by malaria, it is a leading cause of illness and death [2]. In areas with high prevalence, the most vulnerable groups are young children, who have not yet developed immunity to malaria, and pregnant women, whose immunity has been decreased due to pregnancy [3]. In low-prevalence areas, the women generally have developed no immunity to malaria [4]. Malaria infection is the major cause of maternal anemia, premature delivery, and fetal loss [3,4]. For equal exposure, adult men and women are equally vulnerable to malaria infection, but pregnant women are at greater risk of malaria infection [4,5].

In 2018 and 2020, available statistics showed that more than 228 million malaria cases with 405,000 deaths and 241 million cases of malaria with 627,000 deaths were observed, respectively [6,7]. Of these estimates, approximately 93% of malaria cases and 94% of the malaria deaths occurred in Africa and malaria is considered one of the main indirect reasons for maternal and infant mortality [6]. The WHO has found that the African region carries a disproportionately high share of the global malaria burden, and that the region was home to 95% of malaria cases and 96% of malaria deaths in 2020 [1,8]. A new cause-of-death methodology was applied to 32 countries in sub-Saharan Africa and it was found that this region shoulders about 93% of all malaria deaths globally [1,9]. The case fatality is quite dominant among pregnant women and children under five years of age as these are the cohorts with the higher risk of infection and adverse effects [10] For instance, an estimated 25 million pregnancies in sub-Saharan Africa are at risk of malaria, with serious implications for both mother and fetus (e.g., maternal anemia, stillbirths, low birth weight, and intrauterine growth restriction) [11].

If appropriately and regularly used, insecticide-treated nets (ITNs) have been recognized as a cost-effective malaria-prevention intervention that decreases malaria morbidity and mortality by decreasing the contact and bites of mosquitoes [12,13] Previous research has shown that ITN usage is the most cost-effective intervention to reduce malaria transmission in developing countries [14,15]. Empirical evidence suggests that ITNs minimize malaria deaths from all causes of mortality in malaria by nearly 20% [16].

Studies suggest that ITN utilization among pregnant women in sub-Saharan countries is quite low [17,18,19], with cited factors accounting for poor ITN utilization including poor educational and knowledge levels, ITN accessibility, sufficiency, quality, physical condition, maintenance, replacement, and effectiveness [20,21]. However, the effect of these socio-demographic factors may vary across countries and within and between contexts and over time.

Malaria continues to be a major public-health problem in Ethiopia which is one of the countries with the highest prevalence of malaria among pregnant women. Approximately 68% of Ethiopian people live in malaria-risk areas, with about 75% of the landmass of Ethiopia being malaria-endemic and it is one of the most malaria-prone countries in Africa [22]. Given that pregnant women and their unborn children are a vulnerable group and susceptible to malaria and its adverse effects, providing current empirical information on ITN utilization and its related factors may guide authorities in planning appropriate interventions or programs aimed at contributing to national malaria-prevention- and control-policy development. There has been no study conducted at the national level regarding ITN utilization among pregnant women in Ethiopia that considers ITN utilization at the regional and household level. Therefore, based on the data obtained from the 2016 Ethiopian Demographic and Health Survey (EDHS) this study aimed to assess the variation in ITN usage and its associated factors among pregnant women in Ethiopia.

## 2. Materials and Methods

### 2.1. Data Source

The data used for this study were obtained from the 2016 EDHS. Based on a nationally representative sample, the survey was conducted from 18 January 2016 to 27 June 2016, and provided national, regional, and rural estimates [23]. In 2016, the EDHS sample was stratified and selected in two stages. Each region was stratified into urban and rural areas, yielding 21 sampling strata. Samples of EAs (enumeration areas) were selected independently in each stratum in the two stages. A total of 645 EAs was selected in the first stage (202 in urban areas and 443 in rural areas), with probability proportional to EA size (based on the 2007 population and housing census) and independent selections. In the second stage of selection, a fixed number of 28 households per cluster were selected with an equal probability of systematic selection [23]. Details of the EDHS methodology and sampling have been published in the EDHS report, which is also available online at https://dhsprogram.com/publications/publication-fr328-dhs-finalreports.cfm?cssearch=351226_1 (see Supplementary Materials: accessed on 8 January 2022).

### 2.2. Study Population

The target population was women who were pregnant during the survey time. All women who were usual members of the selected households and those who spent the night before the survey in the selected households were eligible to be interviewed in the survey. A total weighted sample of 1122 pregnant women was included in the final analysis of this study.

### 2.3. Study Variables

#### Outcome Variable

Pregnant women’s usage of ITNs was the outcome variable of the study. It was measured as dichotomous as follows:

(1) $$\mathrm{Pregnant}\;\mathrm{women}’s\;\mathrm{usage}\;\mathrm{of}\;\mathrm{an}\;\mathrm{ITN}\;\left(Y_{i}\right)=\{\begin{array}{c} 0:\;woman\;slept\;under\;a\;net\;\\ \;1:\;woman\;did\;not\;sleep\;under\;a\;net \end{array}$$

### 2.4. Independent Variables

#### 2.4.1. Individual Level Variables

The ages of the women (15–24, 25–34, 35–44, 45–49), their educational level (no education, primary, secondary, higher), their working status (working, not working), their wealth index (poorest, poorer, middle, richer, richest), the sex of the household head (male, female), the household size (less than five, five or more), the current marital status of women (never in a union, married/living with a partner, widowed/divorced/separated), and belief that mosquito bites cause malaria (no, yes) were the individual-level variables.

#### 2.4.2. Community Level Variables

Place of residence (urban, rural).

#### 2.4.3. Regional Level Variables

Region (Tigray, Affar, Amhara, Oromia, Somalia, Benishanshangul-Gumuz, SNNPR, Gambela, Harari, Dire Dawa, Addis Ababa).

### 2.5. Data Analyses

Bivariate and multilevel logistic regression analysis were conducted. The variables with *p*-values less than 0.25 in the bivariate analysis were considered to be relevant for the multilevel analysis. A three-level multilevel logistic regression analysis was applied to assess the effects of individual-, community-, and regional-level factors on ITNs usage among pregnant women in Ethiopia. The three-level multilevel logistic regression model was as follows:

(2) $$Y_{ijk}=\beta_{0}+U_{0jk}+\varepsilon_{0ijk}$$

where $Y_{ijk\;}$ is the *i*th pregnant woman in the *j*th community and the *k*th is those who slept under a net, $\beta_{0},\;\beta_{1},\;\beta_{2},\ldots ,\;\beta_{p}$ are the standard logistic regression parameters of the variables associated with ITN usage among the pregnant women, $X_{1}$ $X_{1},\;X_{2,\ldots ,}X_{p}$ are independent variables related to the pregnant women’s usage of an ITN, and $U_{ojk}$ is the random effect at levels two and three.

#### 2.5.1. Multilevel Logistic Regression Intercept-Only Model

An intercept-only model is a model without explanatory variables and it serves as a point of reference with which other models are compared. The three-level intercept-only multilevel logistic regression model is expressed as follows:

(3) $$Y_{ijk}=\beta_{0}+U_{0jk}+\varepsilon_{0ijk}$$

where the index *i* indicates individual women, *j* indicates community-level factors, *k* indicates regional level factors, $U_{0jk}$ is level two and three errors, $\varepsilon_{0ij}$ is a level one error, $\beta_{0}$ is the overall average of pregnant women’s usage of ITNs, and $Y_{ijk}$ is the pregnant *i*th woman’s usage of an ITN in the *j*th community and *k*th region.

#### 2.5.2. Random-Intercept Multilevel Logistic Regression Model

In the random-intercept model, the intercept is the only random effect, meaning that the groups differ with respect to the average value of the ITN usage among the pregnant women, but the relationship between factors and response variables cannot differ between groups. The random intercept model expresses the log-odds, (i.e., the logit of π) as a sum of a linear function of the explanatory variables. That is,

(4) $$\log\left(\frac{\pi }{1-\pi }\right)=\beta_{0j}+\beta_{1}X_{i11}+\beta_{2}X_{i22}+,\ldots ,\beta_{j}X_{ijk\;\;}$$

where π is the probability that the pregnant woman slept under a net and the intercept term $\beta_{0j}\;$ is assumed to vary randomly and is given by the sum of intercept $\beta_{0}$ and group-dependent deviations $U_{ujk}$. That is $\beta_{0j}=\beta_{0}+U_{ojk}$.

#### 2.5.3. Multilevel Logistic Regression Random-Coefficient Model

In this model, the coefficients of the explanatory variables are considered as random.

(5) $$\log\left(\frac{\pi }{1-\pi }\right)=\beta_{oj}+\beta_{1j}X_{i11}+\beta_{2j}X_{i22}+,\ldots ,\beta_{Pj}X_{iPj\;\;\;}+U_{oj}$$

Letting $\beta_{oj}=\beta_{o}+U_{ojk},\;$ and $\beta_{hj}=\beta_{h}+U_{hjk}$ *h* = 1,2,…,*k*. We get

(6) $$\log\left(\frac{\pi }{1-\pi }\right)=\beta_{o}\overset{k}{\underset{h=1}{\sum }}\beta_{h}x_{hij}+U_{ojk}+\overset{k}{\underset{h=1}{\sum }}U_{hjk}x_{hijk}$$

where $\beta_{o}\overset{k}{\underset{h=1}{\sum }}\beta_{h}x_{hij}$ is the fixed part of the model, $U_{ojk}+\overset{k}{\underset{h=1}{\sum }}U_{hjk}x_{hijk}$ is the random part of the model, π is the probability that the pregnant woman slept under a net, and the intercept term $\beta_{0j}\;$ is assumed to vary randomly and is given by the sum of intercept $\beta_{0}$ and group-dependent deviations $U_{ujk}$. That is $\beta_{0j}=\beta_{0}+U_{ojk}$.

#### 2.5.4. Parameter Estimation of Multilevel Logistic Regression Analyses

Multilevel models are also generally estimated using maximum likelihood methods. Combining multilevel and generalized linear models leads to complex models and estimation procedures. The most frequently used methods are based on a first- or second-order Taylor expansion of the link function. When the approximation is around the estimated fixed part, it is called the marginal quasi-likelihood (MQL) and when it is around an estimate for the fixed plus random part, it is called penalized or predictive quasi-likelihood [24,25].

#### 2.5.5. Measures of Variation

Intra-cluster correlation (*ICC*) measures the proportion of variance in the outcome explained by the grouping structure and is determined as follows:

(7) $$\begin{array}{cc} ICC\left(Community\right)=\frac{\delta^{2}{}_{community}}{\delta^{2}{}_{community+\delta^{2}{}_{Region}}+\frac{\pi^{2}}{3}} & \mathrm{ICC}\;\mathrm{attributable to level 2} \end{array}$$

(8) $$\begin{array}{cc} ICC\left(Region\right)=\frac{\delta^{2}{}_{Region}}{\delta^{2}{}_{community+\delta^{2}{}_{Region}}+\frac{\pi^{2}}{3}} & \mathrm{ICC}\;\mathrm{attributable to level 3} \end{array}$$

where $\frac{\pi^{2}}{3}\;$ denotes the variation of lower (individual) level unit, $\delta^{2}{}_{community}$ denotes the variation between communities, and $\;\delta^{2}{}_{Region\;}$ denotes the variation between regions.

#### 2.5.6. Model Comparisons 

In this study, model comparisons were made between four models. Model I was the empty model or null model, Model II included individual-level factors, Model III included community-level factors, and Model IV accounted for both individual- and community-level factors of the study. The model with a small value of *AIC* is the optimal model, which means the best data-fitted model and the model which has few parameters to be estimated [26].

*AIC* is defined as:

(9) AIC=−2ln(likelihood)+2k

where *k* is the degrees of freedom calculated as the rank of a variance–covariance matrix of the parameters.

#### 2.5.7. Bayesian Information Criterion (*BIC*) 

The Bayesian information criterion (*BIC*) is a criterion for model selection among a finite set of models [27]. It is based, in part, on the likelihood function, and is closely related to the Akaike information criterion (*AIC*). It is given as:

(10) BIC=−2LL+log(N)k

where *k* is the degrees of freedom calculated as the rank of a variance–covariance matrix of the parameters and *N* is the number of observations used in the estimation or, more precisely, the number of independent terms in the likelihood.

Thus, based on these two commonly used model-comparison approaches, the model with smallest *AIC* or *BIC* become the best data-fitted model.

## 3. Results 

### 3.1. Descriptive Statistics on the Sample Characteristics

Of the 1122 women included in the study, 467 (41.63%) did not sleep under a net.

The study found that 58.37% of the pregnant women slept under a net and 41.63% of pregnant women did not sleep under a net during the survey time. Table 1 shows that of the 1122 pregnant women included in the study, 197 (17.55%) resided in urban areas, and 925 (82.44%) resided in rural areas. About 698 (62.2%) of the women had no education, 299 (26.7%) had primary education, 74 (6.6%) had secondary education, and 51 (4.5%) had a higher education. Of the women, 548 (48.8%) were aged between 15 and 24, 507 (45.2%) were aged between 25 and 34, and 67 (6%) were aged between 35 and 44.

### 3.2. Bivariate Analysis

From Table 1, variables with a *p*-value less than 0.25 were candidate variables for the multilevel logistic regression analysis. The age, educational level, wealth index, current marital status, region, place of residence, sex of the household head and belief that mosquito bites cause malaria had a *p*-value of less than 0.25. These were the candidate variables for the multilevel analysis but women’s working status and household size were not candidate variables and were not considered for further analysis.

### 3.3. Model Comparisons 

From Table 2, the *AIC* and *BIC* for pregnant women’s usage of ITNs in Model IV (individual-, community-, and regional-level factors) was small compared to the other three models. Thus, Model IV (individual-, community-, and regional-level factors) was the best-fitted to the study data.

### 3.4. Result of Multilevel Logistic Analysis

Before considering the multilevel analysis with all independent variables, the authors tried to identify whether there was heterogeneity in the pregnant women’s usage of ITNs at the community and regional levels by using the null (empty) model. This model contained the response variable and the community- and regional-level variables.

Table 3 shows that the estimated variance in the pregnant women’s usage of ITNs for model I was 0.767 and 0.345, respectively, for the community level and the regional level. This suggested that there was a significant variation of pregnant women’s usage of ITNs between communities and between regions. The *ICC* for the intercept-only model (Model I) was 0.174 at the community level and 0.078 at the regional level. This shows that about 17.4% and 7.8% of the variation in pregnant women’s usage of ITNs was due to variations at the community level and regional level, respectively.

From the final model (model IV), region, place of residence, belief that mosquito bites cause malaria, and the women’s wealth index, educational level, and age were the significant factors in their usage of ITNs. The intra-community correlation coefficient of pregnant women’s usage of ITNs in model IV was estimated to be 0.264. This means that about 26.4% of the total variability in pregnant women’s usage of ITNs was due to differences across the community (place of residence), with the remaining 73.6% of variation in pregnant women’s usage of ITNs attributable to individual differences. The intra-region correlation coefficient of pregnant women’s usage of ITNs in model IV was estimated to be 0.19. This means that 19% of the total variability in pregnant women’s usage of ITNs was due to differences across the region, with the remaining 81% of variation in pregnant women’s usage of ITNs attributable to individual differences.

Controlling other variables in the model as a constant, the odds of usage of ITNs among pregnant women aged between 25 and 34 were 13.8% more than among those aged between 15 and 24. The odds of usage of ITNs among pregnant women with primary, secondary, or higher educational levels were 13.31%, 13.46%, and 12.25%, respectively, more than among pregnant women with no education. The odds of usage of ITNs for poorer- and middle-wealth-index women were 16.88% and 17.54%, respectively, more than among the poorest pregnant women. The odds of usage of ITNs among pregnant women who believed that mosquito bites cause malaria were about 14.9% more than among those who believed mosquito bites do not cause malaria. The odds of usage of ITNs among pregnant women who resided in rural areas were 12.48% more than those who resided in urban areas. Controlling other regions in the variable as a constant, the odds of usage of ITNs among pregnant women in the Amhara, Oromia, Somali, SNNP, and Dire Dawa regions were about 14.8%, 25.4%, 24.5%, 52.7%, and 48.9% more, respectively, than those from the Tigray region.

## 4. Discussion

Based on data obtained from the 2016 EDHS, the current study focused on pregnant women’s usage of ITNs in Ethiopia and, using bivariate and multilevel logistic regression analysis, identified the factors associated with usage of the nets. The study found that 58.37% of pregnant women slept under a net. This result is consistent with previous studies [28,29,30,31,32]. Women’s educational level was associated with ITNs usage among pregnant women; those with secondary or higher educational levels were more likely to sleep under a net than those who had no education. A similar study conducted in Nigeria [33] confirmed this finding and argued that based on the demographic profile, the majority of women had either attained a secondary or higher educational level. The high literacy level, no doubt, played a significant role in their knowledge because educated women are able to comprehend the information provided by the newspapers and other mass media. Education promotes empowerment and ensures development benefit through a continuous learning process, so that the pregnant women learn more about ITNs usage [34]

We also found that the richest women were more likely to use ITNs during pregnancy than the poorest women. However, a study conducted in Kinshasa showed that the poorest women were more likely to use a net during pregnancy than other women [35]. Owning more than one net was associated with a nearly two-fold increased likelihood of using a net in pregnancy [36]. This finding is possibly due to pressure caused by poverty and a low incidence of malaria and shows that intensified education among the poorest women is crucial because they may have ITNs but may use them for other purposes or even sell them to make a living due to unavailability of food and other necessities of their families [37].

The ages of the pregnant women were significant to their usage of ITNs. Pregnant women aged 45 years and above were less likely to use ITNs than younger women. There was a similar finding in a study conducted in southern Ethiopia [38]. Place and region of residence were significant factors in pregnant women’s usage of ITNs. Specifically, pregnant women from rural areas were less likely to use ITNs than women from urban areas. A similar study argued that relative to rural women, women of urban residence were more likely to use ITNs [39]. This is perhaps because they are more familiar with the education and health systems and how the nets are used. It is well established in literature that ownership of ITNs does not necessarily translate into utilization. Given that most health-care facilities in sub-Saharan Africa are predominantly concentrated in urban locations, being prompted and motivated to use their ITNs by healthcare professionals is a necessity [39,40].

Other findings indicate that women in rural areas are more likely to use ITNs. This outcome shows that rural residents in Ethiopia have a higher chance of using ITNs than urban residents following a national mass-distribution exercise. This finding is similar to that of other studies in Ghana and Cameroon [41,42]. In contrast, Ameyaw et al. [31] noted that rural residents in Uganda have a lower chance of using ITNs than urban residents following a national mass-distribution exercise.

The regional factor was another significant factor in ITNs utilization. This is because of malaria-endemic areas that might have forced households to use ITNs for fear of malarial infection. The sex of the household head was not significant in this study. This contradicted the studies conducted by Krah et al. and Renne et al. [43,44]. The ratio of ITNs to household members is the reason for this outcome. With fewer ITNs, and taking into account the traditional messaging on ITNs use by women and children under five, men should give preference to females when there are not enough ITNs available. The reason for this is that women often sleep under the same net as the children under five, who are the most vulnerable. This explanation contradicts some suggestions that give males in the household the power of decision-making, and the belief that males will use ITNs if there are not enough for all household members. The marital status of women was also not a significant factor in ITNs use, a finding similar to that of a study conducted in Nigeria by Onoriode Ezire et al. [45].

Other studies have shown that interpersonal communication and community engagement are excellent ways to build skills and increase knowledge [46,47]. Although ownership of a net is very significant, we did not find a significant relationship between the number of nets owned, the length of time the household had owned the net, and its actual use. Accordingly, having the right information and skills are more important than owning a large number of nets.

## 5. Conclusions

The study found that the prevalence of ITNs usage is relatively low in Ethiopia. The region, place of residence, belief that mosquito bites cause malaria, and wealth index, educational level, and age of the women were the significant factors associated with pregnant women’s usage of ITNs. In light of these findings, it is necessary to analyze and improve current ITNs inventions. Specifically, awareness creation by the community and health-extension workers of the importance of ITNs usage in Ethiopia is necessary. The Ethiopian government should provide awareness to pregnant women across primary healthcare centers and expand public knowledge on the importance of ITNs through targeted and multimedia approaches.

It was found that the prevalence of ITNs use in the study area was lower than the national target and its main barriers were misconceptions and misperceptions regarding ITNs and malaria. Therefore, improving ITNs utilization would require reversal of the community’s misconceptions about malaria and ITNs through information, education, communication, and behavioral change.

## Figures and Tables

**Table 1:** Result of descriptive and bivariate analyses.

		Pregnant Women’s ITN Usage			
Variables	Categories	Women Who SleptUnder a Net	Women Who Did Not Sleep under a Net	Total	*p*-Value $\mathrm{For}\;X^{2}$
Place of residence	Urban	138	59	197 (17.55%)	<0.001
	Rural	517	408	925 (82.45%)	
Region	Tigray	91	14	105 (9.56%)	<0.001
	Afar	34	46	80 (7.13%)	
	Amhara	56	45	101 (9%)	
	Oromi	74	61	135 (12.03%	
	Somalia	162	91	253 (22.55%)	
	Benishangul-Gumuz	37	55	92 (8.2%)	
	SNNPR	107	86	193 (17.02%)	
	Gambela	21	8	29 (2.58%)	
	Harari	23	13	36 (3.21%)	
	Addis Ababa	46	8	54 (4.8%)	
	Dire Dawa	4	40	44 (3.92%)	
Educational level	No education	393	305	698 (62.2%)	0.001
	Primary	170	129	299 (26.7%)	
	Secondary	56	18	74 (6.6%)	
	Higher	36	15	51 (4.5%)	
Wealth index	Poorest	197	176	373 (33.24%)	0.014
	Poorer	131	86	217 (19.34%)	
	Middle	90	61	151 (13.46%)	
	Richer	69	66	135 (12.03%)	
	Richest	168	78	246 (21.93%)	
Age	15–24	301	247	548(48.8%)	<0.001
	25–34	301	206	507 (45.2%)	
	35–44	53	14	58 (6%)	
Current marital status	Never in a union	2	0	2 (0.2%)	0.0018
	Married	643	455	1098 (97.86%)	
	Separated	10	12	22 (1.94%)	
Working status	Not working	461	316	777 (69.25%)	0.450
	Working	194	151	345 (30.75%)	
Belief that mosquito bites cause malaria	No	397	279	676 (60.3%)	0.0024
	Yes	258	188	446 (39.7%)	
Household size	<5	2	5	7 (0.62%)	0.785
	≥5	653	462	1115 (99.38%)	
Sex of household head	Male	535	350	885 (78.87%)	<0.001
	Female	120	117	237 (21.13%)	

**Table 2:** Model comparison.

Response	Model Comparison Criteria	Null Model (Model I)	Individual-Level Factors (Model II)	Community-Level Factors (Model III)	Individual- and Community-Level Factors (Model IV)
Pregnant women’s usage of ITNs	*AIC*	1436.051	1431.974	1414.482	1389.77
	*BIC*	1532.432	1479.779	1446.097	1438.32

**Table 3:** Results of multilevel logistic regression random-coefficient model of pregnant women’s usage of ITNs.

Variable	Null Model (Model I)	Model II	Model III	Model IV
**Fixed effect**	AOR (95% CI)	AOR (95% CI)	AOR (95% CI)	AOR (95% CI)
Constant				0.67 (0.44–1.83)
**Age**				
15–24 (reference category)				
25–34		0.782 (0.533–1.46)		1.38 (1.024–3.11) **
35–44		0.351 (0.187–1.45)		0.78 (0.246–1.32)
45 and above		0.867 (0.125–2.79)		0.35 (0.110–1.76)
No education (reference category)				
Primary		0.521 (0.290–1.88)		1.331 (1.026–1.94) *
Secondary		0.657 (0.335–1.28)		1.346 (1.314–1.59) *
Higher		0.761 (0.438–1.99)		1.225 (1.14–1.77) *
**Wealth index**				
Poorest (reference category)				
Poorer		0.338 (0.136-.87) *		1.688 (1.237–3.44) *
Middle		0.651 (0.366–2.19]		1.754 (1.396–2.74) *
Richer		0.846 (0.761–1.57)		1.142 (0.66–1.49)
Richest		0.289 (0.167–0.42) *		1.021 (0.992–1.534)
**Sex of household head**				
Male (reference category)				
Female		1.440 (1.338–2.67) *		0.62 ([0.356–1.33)
**Current marital status**				
Never in union (reference category)				
Married/living with partner		0.342 (0.153–1.466)		0.49 (0.313–1.55)
Widowed/divorced/separated		0.130 (0.065–1.51)		0.193 (0.064–2.91)
**Belief that mosquito bites cause malaria**				
No (reference category)				
Yes		1.384 (0.766–1.93)		1.49 (1.224–1.854) *
**Place of residence**				
Urban (reference category)				
Rural			1.158 (0.531–1.73)	1.248 (1.0145–2.97) *
**Region**				
Tigray(reference category)				
Afar			0.94 (0.641–3.24)	0.87 (0.65–9.46)
Amhara			1.49 (1.159–2.53) *	1.48 (1.160–2.52) *
Oromia			2.52 (2.981–4.21) *	2.54 (2.987–4.199) *
Somali			2.451 (1.352–5.35) *	2.452 (1.354–5.33) *
Benishangul-Gumuz			2.94 (0.137–4.748]	2.941 (0.945–4.743)
SNNPR			5.26 (3.681–9.58) *	5.27 (3.684–9.54) *
Gambela			3.24 (0.686–9.323)	3.22 (0.685–9.320)
Harari			2.868 (0.773–8.59)	4.865 (0.776–18.55)
Addis Ababa			5.89 (0.261–17.479)	5.87 (0.264–17.476)
Dire Dawa			4.99 (2.72–7.23) *	4.89 (2.76–7.03) *
**Random effect**				
Variance (community)	0.767 (0.588–976)	0.601 (0.322–3.89)	0.927	1.607
*ICC* (community)	0.174	0.143	0.194	0.264
Variance (region)	0.345 (0.177–514)	0.299 (0.151–1.96)	0.540	1.182
*ICC* (region)	0.078	0.071	0.113	0.19

* *p* < 0.05, ** *p* < 0.01; AOR=adjusted odds ratio; CI=confidence interval

## Data Availability

The dataset used for the study is available at: https://dhsprogram.com/data/available-datasets.cfm. Article, Communication, Brief Report, Case Report, Proceedings, Data Descriptor, Project Report (accessed on 8 January 2022).

## Supplementary Materials

Supplementary information about the DHS data usage and ethical standards are available at http://goo.gl/ny8T6X (accessed on 8 January 2022).
